# Muscle-Specific Kinase Myasthenia Gravis

**DOI:** 10.3389/fimmu.2020.00707

**Published:** 2020-05-08

**Authors:** Lucia S. Borges, David P. Richman

**Affiliations:** Department of Neurology, University of California, Davis, Davis, CA, United States

**Keywords:** myasthenia gravis, muscle specific kinase, neuromuscular junction, pathogenesis, treatment, animal models, review

## Abstract

Thirty to fifty percent of patients with acetylcholine receptor (AChR) antibody (Ab)-negative myasthenia gravis (MG) have Abs to muscle specific kinase (MuSK) and are referred to as having MuSK-MG. MuSK is a 100 kD single-pass post-synaptic transmembrane receptor tyrosine kinase crucial to the development and maintenance of the neuromuscular junction. The Abs in MuSK-MG are predominantly of the IgG4 immunoglobulin subclass. MuSK-MG differs from AChR-MG, in exhibiting more focal muscle involvement, including neck, shoulder, facial and bulbar-innervated muscles, as well as wasting of the involved muscles. MuSK-MG is highly associated with the HLA DR14-DQ5 haplotype and occurs predominantly in females with onset in the fourth decade of life. Some of the standard treatments of AChR-MG have been found to have limited effectiveness in MuSK-MG, including thymectomy and cholinesterase inhibitors. Therefore, current treatment involves immunosuppression, primarily by corticosteroids. In addition, patients respond especially well to B cell depletion agents, e.g., rituximab, with long-term remissions. Future treatments will likely derive from the ongoing analysis of the pathogenic mechanisms underlying this disease, including histologic and physiologic studies of the neuromuscular junction in patients as well as information derived from the development and study of animal models of the disease.

## Introduction

Myasthenia gravis (MG) is an autoimmune disease of the neuromuscular junction synapse (NMJ) characterized by weakness that worsens with continued muscle work and improves with resting of the involved muscle(s). Non-immune genetic diseases of this synapse, referred to as congenital myasthenic syndromes, produce similar symptoms (1, 2). For MG, the distribution of weakness is distinctive, involving primarily the extraocular muscles. In ocular MG, involvement is limited to these muscles. In more severe cases (generalized MG), the pontine- and bulbar-innervated muscles and the respiratory muscles are commonly also affected. Least frequently involved are the extremity muscles.

Most MG patients have circulating antibodies (Abs) to the NMJ postsynaptic neurotransmitter receptor, nicotinic acetylcholine receptor (AChR), AChR-MG (3, 4). The pathogenic role of these Abs has been demonstrated by induction of MG in experimental animals by both passive transfer of MG serum Abs (5) or anti-AChR monoclonal Abs (mAbs) (6–8) and by active immunization with purified AChR (9). For both AChR-MG and its experimental models, the Abs induce a destructive inflammatory attack on the AChR-containing postsynaptic membrane (10–13). In generalized MG, AChR Abs are present in 90 percent of patients. The remaining cases were initially designated as seronegative MG.

The earliest studies of these AChR Ab-negative MG cases failed to identify clinical or electrophysiologic features that distinguished it from AChR-MG. In 2001, Hoch and coworkers identified Abs to a different postsynaptic membrane protein, muscle-specific kinase (MuSK), present in the sera of 30–50 percent of seronegative MG patients (14). Once this group of MuSK-Ab-positive MG patients (MuSK-MG) was identified, clinical characteristics of MuSK-MG were discerned that distinguish it from AChR-MG, suggesting that MuSK-MG is a distinct autoimmune disease. Most striking is muscle wasting in many of the affected muscles. Although MuSK-MG is also an Ab-mediated disease, inflammatory damage to the NMJ does not occur. In fact, the majority of the Abs are of the IgG4 immunoglobulin subclass, which is characterized partly by inability to activate complement or bind to Fc receptors. The proposed mode of action of these auto-Abs is blockade of the normal function of MuSK.

Many of the standard treatments of AChR-MG are of limited effectiveness in MuSK-MG, including thymectomy and cholinesterase inhibitors. Therefore, current treatment involves immunosuppression, primarily by corticosteroids or B cell depletion agents.

Since the initial identification of MuSK-MG, a number of experimental animal models of this disease have been developed. As is the case in AChR-MG, careful analyses of both the human disease and the animal models have led to the determination of the pathogenic mechanisms underlying this disease. Such information has the potential for the development of improved treatments of MuSK-MG and similar diseases.

## MuSk and the Neuromuscular Junction

**FIGURE 1:**
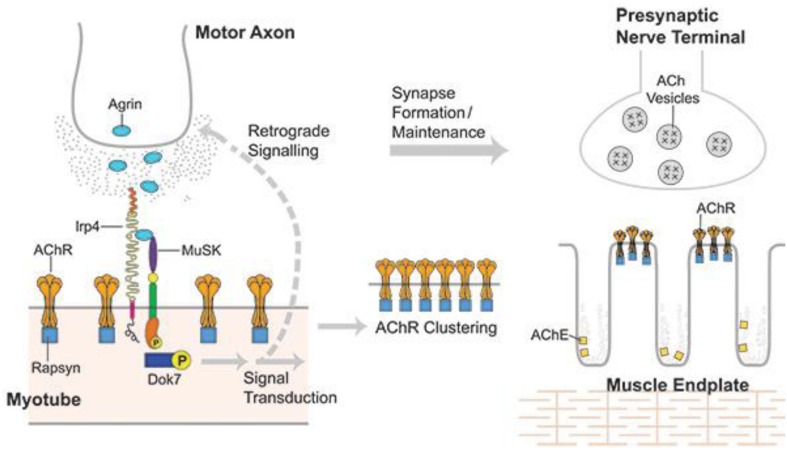
Developing NMJ: The motor axon growth cone releases agrin into the intercellular matrix when it reaches a developing myotube. Agrin binds lrp4 and the complex binds MuSK resulting in activation of MuSK, which self-phosphorylates and then initiates a series of phosphorylations beginning with Dok7 and ending with rapsyn and 8 subunit of AChR. This process induces dense AChR clustering, the first step in the development of both the postsynaptic and presynaptic portions of the mature NMJ. From Richman (66) with permission.

**FIGURE 2:**
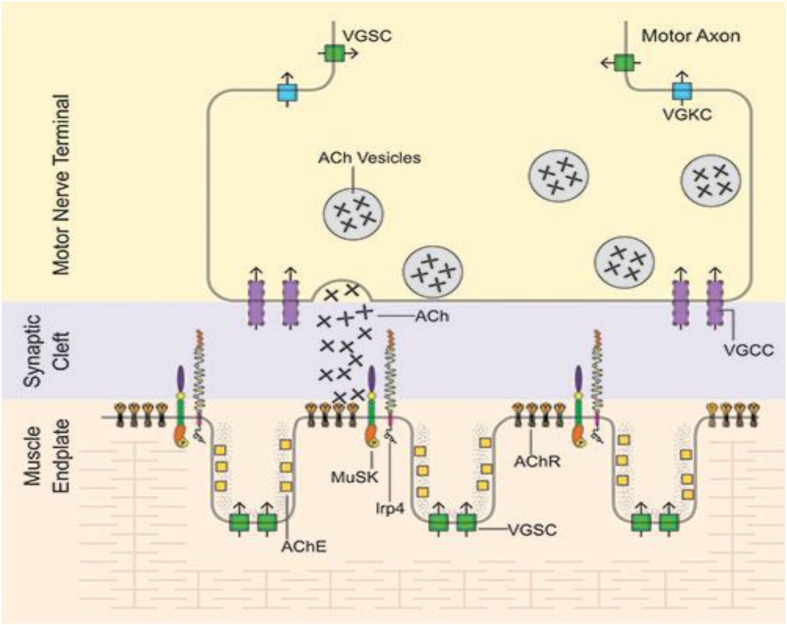
Mature NMJ: Motor axon action potentials reach the motor nerve terminal leading to release of vesicles of acetylcholine (ACh), which diffuses across the synaptic clef to bind to the tightly packed acetylcholine receptors (AChR) located on the peaks of the folds of the endplate membrane. After AChR activation, ACh is then hydrolyzed by acetylcholinesterase (AChE) in the muscle basal lamina. VGSC, voltage-gated sodium channels; VGKC, voltage-gated potassium channels; VGCC, voltage-gated calcium channels. From Richman (66) with permission.

**FIGURE 3:**
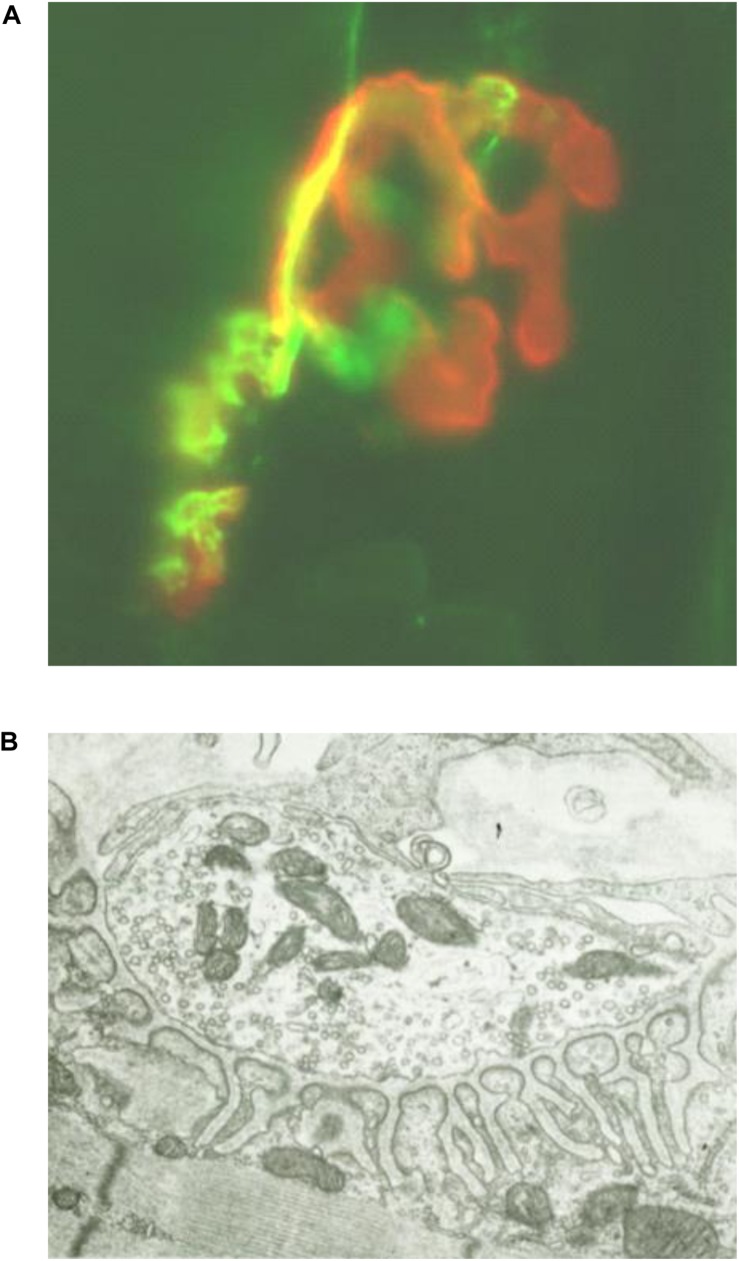
**(A)** Photomicrograph (x80) of longitudinal frozen section of diaphragm muscle stained immunohistochemically with alpha-bungarotoxin to label AChR (red) and anti-synapsin plus anti-neurofilament Abs to label presynaptic nerve terminals and axons (green), demonstrating pretzel appearance of endplate membrane. **(B)** Electron micrograph (×5000) of transverse section of diaphragm muscle neuromuscular junction demonstrating highly folded endplate membrane. [Modified from (63) with permission].

Muscle specific kinase was identified as a postsynaptic integral membrane protein playing a pivotal role in the development of the NMJ (15–19). This synapse begins to form when the axon growth cone of a developing motor neuron encounters a developing myotube and begins to secrete agrin, a glycoprotein with a laminin-binding domain that anchors it to the extracellular matrix (20–25). Prior to the arrival of the axon, AChRs, which initially are spread diffusely along the myotube, begin to cluster in the central region of the myotube (26, 27). When the axon growth cone eventually encounters this region and secretes agrin (Figure 1) (9, 10), the agrin induces more extensive dense clustering of the AChRs in the postsynaptic endplate membrane, which is the first step in the elaboration of this structure into its adult architecture (Figure 2), including a pretzel-like topographic profile (Figure 3A) and marked folding and specialization of that membrane at the ultrastructural level (Figure 3B) (20–25, 28–34).

Both the initial spontaneous AChR clustering and the agrin-induced effects require the presence of MuSK (23, 35, 36). The paradoxical observation that agrin and MuSK do not bind *in vitro* led to a search for a third (intermediary) protein required for their interaction, which was eventually found and identified as the postsynaptic transmembrane protein low density lipoprotein receptor-related protein 4 (lrp4) (37–39).

The agrin-lrp4-MuSK interaction leads first to MuSK dimerization and then self-phosphorylation. The latter effect initiates a series of intracellular protein phosphorylations mediated through a downstream signal transduction pathway beginning with Dok7 and ending with rapsyn and the β subunit of AChR (40–43). Activation of this pathway results in dense AChR clustering, the first step in the elaboration of the postsynaptic components of the synapse (Figure 2) (44, 45). The AChR clustering also includes MuSK and lrp4 and the other components of the MuSK-associated signaling pathway (21, 46).

Activation of the agrin/lrp4/MuSK pathway leads, as well, to increased expression/synthesis of the components of the pathway and other endplate-specific proteins (by subsynaptic muscle nuclei) (22, 47–49). The induced AChR clustering, and the eventual elaboration of the entire adult postsynaptic endplate structure, involves polymerization of actin leading to the production of an intracellular scaffolding, comprised of a number of proteins, upon which the mature structure of the muscle endplate is formed. This process results in tight packing of the phosphorylated AChRs on the peaks of the synaptic folds opposite the specialized nerve terminal (Figure 3B) (44, 45, 50). This actin/cytoskeletal remodeling is contributed to by a number of other proteins in the MuSK signaling pathway, most prominently cortactin, which when phosphorylated directly enhances further actin polymerization (44, 51). Extracellularly, ColQ, the collagen-like portion of the NMJ enzyme acetylcholinesterase, binds to the extracellular portion of concentrated (clustered) MuSK (52, 53) and also to the extracellular matrix protein perlecan, leading to anchoring of the enzyme to the extracellular matrix at the clustering sites (53).

The agrin/lrp4-induced activation (phosphorylation) of MuSK is also associated with development of the presynaptic portion of the NMJ. MuSK activation initiates a separate (less well understood) retrograde pathway, resulting first in a stop signal terminating the travels of the motor axon (Figure 1) (54, 55). The increased concentration (clustering) of lrp4 at the developing NMJ induced by activation of the MuSK transduction pathway is required for the further development of the axon growth cone into the adult specialized presynaptic nerve terminal. The concentrated lrp4 binds the nerve terminal, but the presynaptic “receptor” for lrp4 and the subsequent developmental steps have not yet been identified (56) (21).

The further maturation of the NMJ and, in particular, the mechanisms involved in the maintenance of the mature NMJ, are even less well understood (33, 55, 57, 58). Maintenance of the NMJ does appear to require MuSK functionality, as demonstrated by the dissolution of the synapse in adult animals (in the absence of inflammation) both in (1) experimental MuSK-MG induced by either passive or active immunization with MuSK (59–63) and (2) in adult animals in which MuSK has been inactivated or knocked down (64, 65).

## MuSK Molecular Structure

**FIGURE 4:**
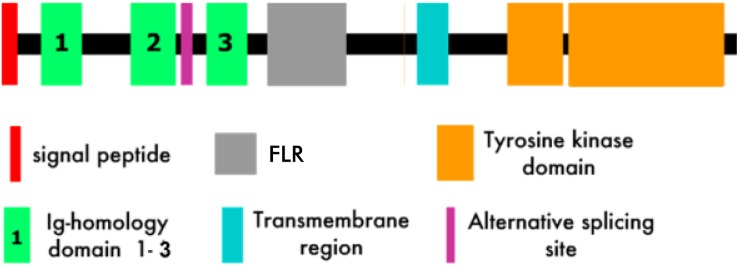
MuSK Structure (Modified from 15). FLR, Frizzled-like region.

Muscle specific kinase is a 100 kD single-pass transmembrane receptor tyrosine kinase with an N-terminal extracellular domain followed by a short transmembrane domain and then a C-terminal cytoplasmic domain (Figure 4) (15, 16, 18, 19). The extracellular domain of MuSK, which is required for interaction with agrin and lrp4, comprises three immunoglobulin (Ig)-like domains (37, 39, 67) followed by a cysteine-rich frizzled-like region (labeled C6-box in Figure 4) (15, 16, 18, 45). The cytoplasmic domain contains the kinase activity and signaling components of the molecule that lead to the development of the postsynaptic apparatus (see above) (45).

The first two extracellular Ig-like domains, which are rigidly joined in a linear array (67), appear to play a dual role in activation of MuSK signaling. First, Ig-1 is crucial for binding to the MuSK ligand, i.e., agrin-associated lrp4 (68). Second, it is the substrate for the dimerization of two MuSK molecules (67). Dimerization is required for MuSK (*trans*) autophosphorylation, the first step in the activation of the MuSK-associated signaling pathway (69, 70). Autophosphorylation, along with binding of Dok7, an intracellular MuSK target, results in full activation of the MuSK kinase activity (71, 72). It is the combination of ligand (agrin/lrp4) binding and the full establishment of its kinase activity that results in the sequence of protein phosphorylations by MuSK that comprise the MuSK-associated signaling pathway that leads first to AChR clustering and subsequently to formation of the mature NMJ. The role of the frizzled-like region, which functions as a receptor for the wnt family of intracellular signaling proteins, is not yet well understood (73–76).

Early studies employing both rat and human MuSK have determined that it is only the extracellular domain of the molecule that is the target of the MuSK Abs in MuSK-MG (see below) (14, 77).

## Anti-MuSK Myasthenia

### Disease Characteristics

The MuSK Ab-positive subgroup of “seronegative patients,” anti-MuSK MG, does have clinical similarities to AChR-MG but tends to differ significantly in exhibiting more focal involvement than AChR-MG, frequently with severe involvement of neck, shoulder, facial and bulbar-innervated muscles, although there is considerable variability from patient to patient (78–82). When the extremities are involved, proximal muscles are more affected than distal ones (83). Unlike AChR-MG, many patients have wasting of these muscles (78, 79, 81, 84–86), and data suggest that this represents a direct myopathy and is not the result of denervation, a point that remains somewhat controversial (86–91).

The demographic characteristics of MuSK-MG differ from AChR MG. In the latter disease, the age/incidence curve is bimodal with a peak in the early 20’s, which is majority female, and with a second peak in the 60’s and 70’s, which is majority male (92–94). In contrast, MuSK-MG tends to occur in the 30’s with very strong female predominance (95). Also, MuSK-MG is highly associated with the HLA DR14-DQ5 haplotype (96).

The restricted HLA (MHC) class II association in MuSK-MG suggests a role for T helper cells in this Ab-mediated disease. A recent study observed antigen (MuSK)-specific T cell responses in cultures of circulating mononuclear cells (MNC) from MuSK-MG patients. These anti-MuSK responses utilized a somewhat limited number of T cell receptor variable region genes (97–99), consistent with a genetically influenced disease-specific T cell response. Also, in contrast to AChR-MG, only rare MuSK MG patients have been found to have thymic lymphoid hyperplasia (97–101).

Remarkably, data concerning NMJ histology and microphysiology in MuSK-MG are very limited. In the three histologic studies available (102–104), only relatively mild abnormalities of NMJ morphology/function were observed. The changes were all postsynaptic, including partially denervated postsynaptic membrane and moderate degeneration of postsynaptic folds (102). One of two microelectrode studies found only postsynaptic abnormalities, marked decrease in miniature endplate potential (MEPP) amplitudes (103). However, the other study found both postsynaptic abnormalities, mild decrease in MEPP amplitudes, and presynaptic abnormalities, reduced levels of presynaptic acetylcholine release (102).

### Anti-MuSK Antibodies in MuSK-MG

Anti-MuSK Abs are detected in 1–10% of patients with MG, 40% of the AChR Ab-negative patients (14, 77, 78, 80, 81, 105). Most of the anti-MuSK Abs belong to the IgG4 immunoglobulin subclass (77, 106), which is unable to either activate complement or induce antigenic modulation (107). However, passive transfer of the IgG4 component of MuSK-MG serum is especially effective in inducing the experimental disease (108).

Abs of the IgG4 subclass behave as if they are functionally monovalent (109). Because of single amino acid differences in the IgG4 heavy chain constant region, the inter-heavy chain disulfide bonds that join the two halves of the immunoglobulin molecule are markedly weakened, leading to frequent separation of the two heavy chains. The resultant “half molecules,” consisting of a single light chain covalently bound to a single heavy chain, can readily bind to a half molecule from another IgG4 Ab to reform a complete Ab molecule, so called “Fab arm exchange” (109, 110). The new Ab molecule is now bispecific, that is each Ab arm binds a different antigen. Such Abs, including IgG4 MuSK Abs, cannot crosslink single antigens and, therefore cannot induce antigenic modulation (which requires antigen crosslinking), a mechanism important in AChR-MG. Also as noted above, these IgG4 Abs are minimally interactive with the innate immune system, in that they are deficient in complement activation and in binding to cell surface Fc receptors (107). Anti-MuSK Abs of the IgG1 subclass, a subclass capable of engaging these components of the innate immune system, are also present in most MuSK-MG patients, but at much lower levels than the IgG4 Abs (111, 112). The role the IgG1 Abs play in MuSK-MG is not known.

Hence, the pathogenic mode of action of the auto-Abs in MuSK-MG differs from that of the AChR Abs in AChR-MG. Rather than inducing destructive damage to the NMJ or antigenic modulation, the anti-MuSK Abs mask the binding sites on MuSK that interact with its binding proteins (ligands), including lrp4/agrin and ColQ, thereby blocking MuSK function (106, 111). Blockade of MuSK ligand binding leads to a reduced postsynaptic density of AChRs and impairs their alignment in the postsynaptic membrane (60).

Most anti-MuSK Abs bind to the Ig-like domains of the extracellular portion of MuSK (Figure 4) (14, 77, 106, 113, 114). In one study of 53 MuSK-MG patients, all had Abs to Ig-like domain 1 and about 50 percent also had Abs to Ig-like domain 2. For female patients, it was rare to have Ab reactivity to domains other than Ig-like domain 1 (113). However, Abs to the frizzled-like domain have been observed in MuSK MG (115).

## Animal Models of Anti-MuSK MG

As noted above, few human studies have addressed directly the pathogenesis of MuSK-MG (102–104). None have observed complement-mediated injury or cellular infiltration of the NMJ. In fact, initially there was controversy concerning the role of anti-MuSK Abs in MuSK-MG pathogenesis (103, 116, 117), in spite of the ability of these Abs to act as MuSK antagonists *in vitro*. On the other hand, experimental studies, involving the induction of experimental models of MuSK-MG, have provided the strongest evidence concerning the pathogenic mechanisms underlying this disease. The data from MuSK-MG animal models induced by both passive and active immunization with MuSK demonstrate the role of the anti-MuSK Abs in the induction of both the weakness and the morphological and physiological NMJ changes observed in MuSK-MG (59, 60, 117–123).

### Passive Immunization Studies

A number of studies have assessed the effect of daily intraperitoneal injections into immunosuppressed mice of very large amounts of IgG (usually 35–50 mg per day) purified from (human) MuSK-MG serum. In one study, injections for 5 days produced reduced neuromuscular transmission but without clinical weakness (118). A second study made use of IgG purified from a severely affected patient injected for 14 days (total of 0.68 g), which resulted in weakness and weight loss. Histologic analysis of these animals found patchy reduction in NMJ AChR staining, reduced registration between nerve terminals and motor endplates (59) and reduced phosphorylation of the downstream components of the MuSK signaling pathway (60). The Abs also produce internalization of MuSK with more rapid degradation leading to reduced endplate MuSK concentrations (60, 117). The marked effectiveness of passive transfer of the IgG4 component of MuSK-MG serum is consistent with the role of these mechanisms in inducing the experimental disease (108). These observations, along with the absence of observed complement-mediated injury, support the hypothesis that the MuSK Abs induce the disease by blocking MuSK signaling *in vivo* with the resultant postsynaptic changes described above, as well as damage to the nerve terminals (see below).

### Active Immunization Studies

In rabbits (117) and, to a lesser extent in mice (118–123), repeatedly immunized with MuSK protein (of human or rat origin) over extended periods of time, induces mild weakness along with mild electrophysiologic evidence of disordered neuromuscular transmission and varying degrees of reduction in motor endplate size. For mice there has been considerable variability among strains in the susceptibility to the active induction of experimental MuSK-MG (118–123). For C57BL6, the distribution of weakness and wasting follows the gradient of normal MuSK expression in individual muscles (120), which somewhat mimics the distribution of muscle involvement in MuSK-MG (see above).

In contrast to the variability in response and the requirement for repeated immunizations in mice, the experimental disease in inbred Lewis rats is highly stereotyped. A single immunization of mouse MuSK ectodomain in adjuvants results in reproducible severe weakness (death within 4 weeks), along with muscle wasting and electrodiagnostic abnormalities typical of MuSK-MG. Histologically, there are extensive postsynaptic and presynaptic changes. The NMJ morphologic findings include fragmented NMJs with varying degrees of postsynaptic muscle end plate destruction, along with abnormal nerve terminals. The presynaptic changes are characterized by reduced terminal size, ongoing terminal degeneration and lack of registration between endplate and nerve terminals. In addition, there is local axon sprouting, and extrajunctional dispersion of cholinesterase activity (61–63).

## Pathogenesis of MuSK-MG

Data from analysis of both MuSK-MG and its animal models described above have contributed to the information concerning the pathogenesis of the human disease. With the identification of MuSK Abs in “seronegative MG” patients, the initial hypothesis was that the MuSK Abs indeed induce the disease. As noted above, at the time, the hypothesis was not universally accepted (103, 116, 117). The alternative hypothesis advanced was that these Abs are an epiphenomenon occurring in parallel with the disease or even occurring as a result of the disease (103, 116). The dual observations (see above) of (1) the ability of IgG isolated from patient serum, when injected into immunosuppressed mice, to induce a disease similar to human MuSK-MG, and (2) immunization of normal animals with purified MuSK protein, leading to the production of MuSK Abs, also results in a disease that is highly similar to human MuSK-MG, together strongly support the hypothesis that the MuSK Abs are the etiologic agents in this disease.

Multiple observations of neuromuscular junction histology from the various animal models, along with the few histologic observations available from MuSK-MG patients, all demonstrating the absence of inflammatory damage, suggests that the innate immune system, especially the complement cascade, does not play a role in this disease. This characteristic distinguishes MuSK-MG from AChR-MG. It should be noted, however, that the data from the mouse passive transfer models employing human anti-MuSK IgG are somewhat confounded by both the necessary pretreatment with immunosuppressive agents, the extremely high doses of human Ig required to induce disease and the necessity in this model for interaction between the injected human IgG and the recipient mouse’s innate immune system. This heterologous interaction, theoretically, may not be strong enough to induce a vigorous inflammatory reaction. On the other hand, for the active immunization model, immunosuppressive agents are not employed and the Ab and the innate immune system components, e.g., complement proteins and inflammatory cells expressing Fc receptors, are autologous (syngeneic) and hence capable of inducing a strong inflammatory response. At least in humans, the absence of observed inflammation in the NMJ, may be the result of the high proportion of IgG4 anti-MuSK Abs in MuSK MG (see above).

Despite the lack of inflammatory damage to the neuromuscular junction in MuSK-MG and its animal models and the theoretical inability of the human anti-MuSK IgG4 to induce antigenic modulation (i.e., increased MuSK turnover), the concentration of AChR in the postsynaptic membrane is reduced, and, to a lesser extent, so is the MuSK concentration (60, 117). The current hypothesis is that the MuSK Abs act as antagonists to the MuSK function as a receptor kinase, with its natural ligand being the agrin/lrp4 complex. *In vitro*, MuSK Abs from MuSK-MG patients and from actively immunized animals block agrin-induced AChR clustering and downstream phosphorylation in muscle cells in tissue culture (14, 59, 106, 111, 124, 125). The latter observations and the development of the above abnormalities in the NMJs in adult animals (with fully developed neuromuscular junctions) in which experimental MuSK-MG is either actively or passively induced, suggests that MuSK plays a crucial role not only in the development of the NMJ, but also plays a role in the maintenance of this synapse in adult animals. Subsequent studies of adult animals in which MuSK has been inactivated or knocked down demonstrated similar NMJ changes (64, 65).

As noted above, presynaptic involvement is another characteristic of MuSK-MG that distinguishes it from AChR-MG. In the latter disease presynaptic dysfunction is absent except in the setting of severe NMJ inflammation (12). Otherwise, presynaptic activity, in fact, is increased (126). Similar to the human disease, the experimental models of MuSK-MG noted above exhibit non-inflammatory structural and functional abnormalities of both presynaptic and postsynaptic portions of the NMJ. The abnormal presynaptic function is most readily observed in the severe form of experimental MuSK-MG that occurs in the active-immunization rat model (63). In that disease model, both the cross-sectional area of individual nerve terminals is reduced as is the total nerve terminal cross-sectional area across the entire NMJ. In addition, the actively induced rat model and the passive transfer mouse model have both demonstrated abnormal registration between the nerve terminals and the muscle endplates. Whether the observed reduction in NMJ cholinesterase activity in MuSK-MG plays a role in the muscle weakness is yet to be determined.

## Treatment of MuSK-MG

Early on, the treatment of seronegative MG simply followed the protocols developed for seropositive MG, i.e., AChR-MG, including use of cholinesterase inhibitors, thymectomy, corticosteroids, plasma exchange and intravenous immunoglobulin and cytotoxic immunosuppressants. However, with the clear identification of the MuSK-MG subset of “seronegative” patients, data began to accumulate that a number of these treatments were ineffective, or even detrimental, in these patients. Thymectomy appears not to play a role in the treatment of this disease (78, 80, 96, 105, 127, 128). Some studies suggest other differences in response to treatment, including observations that MuSK-MG patients may respond poorly to intravenous immunoglobulin (78, 80, 96, 105, 129, 130). There is also a striking absence of improvement with cholinesterase inhibitors (101, 130–133). In fact, a number of MuSK-MG patients worsen in response these agents (101, 131). The latter observation may derive from diminished acetylcholinesterase concentrations at the NMJ, perhaps as a result of MuSK Ab-mediated blockade of ColQ binding to MuSK or Ab-mediated reduction in MuSK concentration (52, 53).

### Current Treatments

#### Corticosteroids

Patients with MuSK-MG respond especially well to high dose corticosteroids (129, 134–136). This appears to be most often true for patients with rapidly progressive or aggressive disease. The effectiveness of steroid treatment, along with the absence of a role for thymectomy and the limited or absent role for cholinesterase inhibitors, make corticosteroids the foundation of the current treatment of this disease. However, about 15 percent of patients treated with high dose corticosteroids do not adequately respond, so called refractory disease. This figure is somewhat higher than the comparable data in AChR-MG (137, 138). Similar to the case in AChR-MG, the significant side effects of these agents often limit their effectiveness. In both diseases, the treatment protocols aim at inducing a remission with high doses, followed by slow tapering of the dose to the lowest effective dose (139). An additional issue in MuSK-MG is the muscle wasting that occurs uniquely in this form of autoimmune MG. In many patients, in spite of the early effectiveness of corticosteroids in inducing a clinical remission, the muscle wasting appears to continue to progress (135).

#### Standard Cytotoxic Immunosuppressants

Cytotoxic agents effective in preventing and treating solid organ transplant rejection have been used as single immunosuppressive agents in the treatment of AChR-MG, including azathioprine, mycophenolate mofetil and cyclosporine, with some efficacy in inducing remission. Their major role in that disease, however, has been as steroid-sparing treatments, that is, effective in facilitating corticosteroid dose tapering (140). Anecdotal data for MuSK-MG suggest that these agents are even less effective in inducing remission than in AChR-MG but, similarly, may be useful as steroid-sparing medications (135, 140, 141).

#### Immune-Directed Biologic Treatments

The B cell depleting agent rituximab, a chimeric anti-CD20 monoclonal Ab, has been especially effective in MuSK-MG. Because of its toxicity profile, including a 1:10,000 risk of the induction of progressive multifocal leukoencephalopathy, the drug has been used primarily in treatment of (steroid)-refractory patients. A significant number of reported studies, all limited by relatively small numbers of subjects, have shown efficacy in this disease (142–146). In a number of cases, treatment led to eventual elimination of the need for other immune-directed treatments, e.g., steroids, and without the necessity for repeated rituximab infusions (143–145). A recent set of clinical guidelines has supported earlier use of this agent when an initial standard treatment does not induce rapid remission (140). One successful protocol is to use two courses of rituximab at a dose of 375 mg per meter squared body surface area weekly for four doses, each course separated by 6 months. A 4-infusion course can then be repeated as needed (143). It appears that adding an infusion 1 month later and another 2 months later improves efficacy even further (147).

#### Short-Term Immune-Directed Treatment

Plasma exchange has been a rapidly effective treatment for active AChR-MG. Intravenous immunoglobulin infusions have been equally effective and somewhat safer (148). Initial studies of plasma exchange in acute MuSK-MG demonstrated its efficacy in this disease (129, 134, 149). Unlike in AChR-MG, the efficacy of intravenous immunoglobulin in MuSK-MG appears to be less than that of plasma exchange (130, 134, 136, 150), but the data supporting the latter statement are much less robust (151, 152).

### Future Treatments: Antigen-Specific Agents

As noted above, the various immune-directed treatments currently in use have been reasonably effective in MuSK-MG. However, these treatments are all limited by their broad effect on the immune system: on both the pathogenic (autoimmune) components and the normally functioning components. As in all other autoimmune diseases, the treatment paradigm is to adjust drug dosages and timing to maximize the effect on the autoimmune portion of the immune response while reducing the “off-target” effect on the remainder of the immune system.

A theoretical means of focusing the treatment on the autoimmune portion is to employ an antigen-specific therapy, that is, one only targeting the attack on the auto-antigen. For an Ab-mediated disease such as MG, this would involve targeting the auto-Abs. One possibly means to accomplish this therapeutic effect involves physical removal of the auto-Abs, for example, by immunoadsorption plasmapheresis. For AChR-MG, such antigen-specific Ab removal employing an affinity column containing AChR antigen as the affinity agent has been examined. To date, this approach has been no more effective than gross removal of all Abs by plasma exchange (153, 154).

An alternative antigen-specific approach is to target the B cells that are secreting the auto-Abs. This approach is currently under study in both AChR-MG and in MuSK-MG, through the use of either genetically engineered Abs or genetically engineered T cells that target the pathogenic autoimmune B cells (155, 156).

## Author Contributions

Both authors listed have made a substantial, direct and intellectual contribution to the work, and approved it for publication.

## Conflict of Interest

LB and DR have received research funding from NINDS (1R21NS104516), Myasthenia Gravis Foundation of America, and Cabaletta Bio Inc. DR is a member of the Advisory Board of Cabaletta Bio Inc.
